# 1,3-Diisopropyl-4,5-dimethyl­imidazolium benzene­sulfonate

**DOI:** 10.1107/S1600536811030236

**Published:** 2011-07-30

**Authors:** Kamal Sweidan, Eyad Mallah, Qutaiba Abu-Salem, Manfred Steimann, Cäcilia Maichle-Mössmer

**Affiliations:** a Department of Chemistry, The University of Jordan, Amman, Jordan; b Faculty of Pharmacy and Medical Science, Petra University, PO Box 961343, Amman 11196, Jordan; c Department of Chemistry, Faculty of Science, University of Al al-Bayt, Al-Mafraq, Jordan; d Institut für Anorganische Chemie der Universität Tübingen, Auf der Morgenstelle 18, 72076 Tübingen, Germany

## Abstract

In the title salt, C_11_H_21_N_2_^+^·C_6_H_5_O_3_S^−^, which has two cation–anion pairs in the asymmetric unit, the two imidazolium cations are linked to two separate acceptor O atoms of one of the benzene­sulfonate anions through aromatic C—H⋯O hydrogen bonds, while the second anion is unassociated.

## Related literature

For the structures of similar compounds, see: Sweidan *et al.* (2009 ▶); Kuhn *et al.* (2007 ▶); Grishina *et al.* (2011 ▶). For the synthesis of the starting material, see: Kuhn & Kratz (1993 ▶).
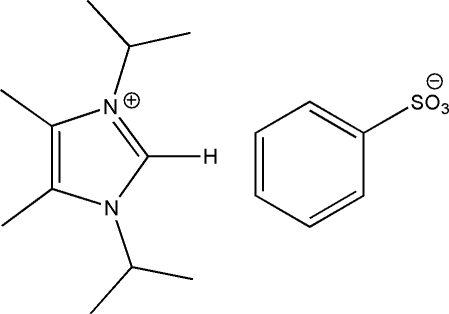


## Experimental

### 

#### Crystal data

C_11_H_21_N_2_^+^·C_6_H_5_O_3_S^−^
*M* *_r_* = 338.46
Triclinic, 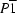

*a* = 8.8691 (9) Å
*b* = 14.1494 (15) Å
*c* = 14.3200 (14) Å
α = 87.082 (8)°
β = 88.326 (8)°
γ = 83.988 (8)°
*V* = 1784.3 (3) Å^3^
*Z* = 4
Mo *K*α radiation
μ = 0.20 mm^−1^
*T* = 173 K
0.40 × 0.15 × 0.15 mm

#### Data collection

Stoe IPDS II CCD diffractometer
25481 measured reflections
7264 independent reflections
5925 reflections with *I* > 2σ(*I*)
*R*_int_ = 0.093

#### Refinement

*R*[*F* ^2^ > 2σ(*F* ^2^)] = 0.048
*wR*(*F* ^2^) = 0.105
*S* = 1.13
7264 reflections
464 parameters
H atoms treated by a mixture of independent and constrained refinement
Δρ_max_ = 0.43 e Å^−3^
Δρ_min_ = −0.27 e Å^−3^

### 

Data collection: *X-AREA* (Stoe & Cie, 2008 ▶); cell refinement: *X-AREA*; data reduction: *X-RED32* (Stoe & Cie, 2008 ▶); program(s) used to solve structure: *SHELXS97* (Sheldrick, 2008 ▶); program(s) used to refine structure: *SHELXL97* (Sheldrick, 2008 ▶); molecular graphics: *SHELXTL* (Sheldrick, 2008 ▶); software used to prepare material for publication: *SHELXTL*.

## Supplementary Material

Crystal structure: contains datablock(s) global, I. DOI: 10.1107/S1600536811030236/zs2131sup1.cif

Structure factors: contains datablock(s) I. DOI: 10.1107/S1600536811030236/zs2131Isup2.hkl

Supplementary material file. DOI: 10.1107/S1600536811030236/zs2131Isup3.cml

Additional supplementary materials:  crystallographic information; 3D view; checkCIF report

## Figures and Tables

**Table 1:** Hydrogen-bond geometry (Å, °)

*D*—H⋯*A*	*D*—H	H⋯*A*	*D*⋯*A*	*D*—H⋯*A*
C1—H1⋯O11^i^	0.95	2.30	3.198 (3)	157
C01—H01⋯O13	0.95	2.25	3.040 (3)	141

Symmetry code: (i) 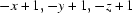.

**Fig. 1.**
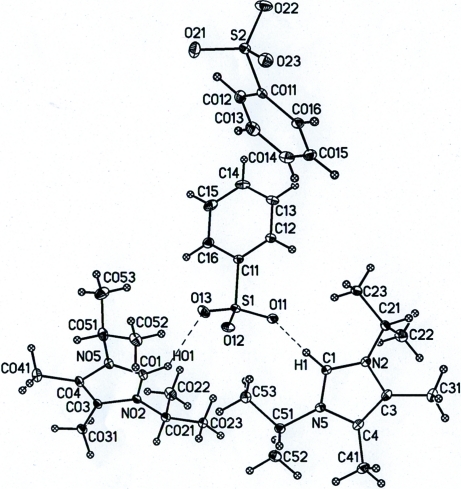
The molecular structure of the two cation–anion pairs in the asymmetric unit of the title molecule showing the atom numbering scheme, with 20% probability displacement ellipsoids for non-H atoms. Inter–species hydrogen bonds are shown as dashed lines

## supplementary crystallographic information

### Comment

Due to the strongly basic character of *N*-heterocyclic carbenes, their reactions with acidic compounds usually produce 2*H*-imidazolium cation pairs. The formation of stable C—H···O bonds between the components of these compounds may act as an additional stabilizing factor in their crystal structures (Kuhn *et al.*, 2007). The structures have shown that these pairs are linked by imidazolium aromatic C—H···O hydrogen bonds as observed commonly in imidazolium salts (Sweidan *et al.*, 2009). However, the crystal structure of 1,3-di-*tert*-butyl-4,5-dimethylimidazolium trifluoromethanesulfonate showed the absence of intermolecular hydrogen bonding between the imidazolium cation and the trifluoromethanesulfonate anion (Grishina *et al.*, 2011).

The structure of the title compound, C_11_H_21_N_2_^+^ C_6_ H_5_O_3_S^-^ (Fig. 1), which has two cation–anion pairs in the asymmetric unit, reveals the presence of intermolecular aromatic C—H···O_sulfonate_ hydrogen-bonding interactions between the two imidazolium cation moieties and two separate oxygen atoms of one of the benzenesulfonate anion moieties (Table 1, Fig. 1). Surprisingly, the second anion is not involved in any intermoleculer hydrogen bonding, which may be attributed to the steric crowding effect of the cation ring.

### Experimental

The title compound was prepared by slow addition of benzenesulfonic acid (0.425 g, 2.67 mmol) to a solution containing 0.481 g (2.67 mmol) of 1,3-diisopropyl-4,5-dimethyl-4,5-dimethylimidazol-2-ylidene (see Kuhn & Kratz, 1993), in 20 ml of dry Et_2_O at -25° C. After stirring overnight at room temperature, the precipitate was filtered off, washed with dry Et_2_O and dried under reduced pressure. Yield: 0.814 g (90%). This solid was recrystallized from CH_3_COCH_3_/ Et_2_O as colorless crystals.

### Refinement

Hydrogen atoms were included in the refinement at calculated positions and some were allowed to refine isotropically while the remainder were included at calculated positions with C—H = 0.95–1.00 Å and with *U*_iso_(H) = 1.2*U*_eq_(aromatic C) or 1.5*U*_eq_(aliphatic C), using a riding-model approximation.

### Crystal data

**Table:**

C_11_H_21_N_2_^+^·C_6_H_5_O_3_S^−^	*Z* = 4
*M**_r_* = 338.46	*F*(000) = 728
Triclinic, *P*1	*D*_x_ = 1.260 Mg m^−^^3^
Hall symbol: -P 1	Mo *K*α radiation, λ = 0.71073 Å
*a* = 8.8691 (9) Å	Cell parameters from 8000 reflections
*b* = 14.1494 (15) Å	θ = 3.2–26.4°
*c* = 14.3200 (14) Å	µ = 0.20 mm^−^^1^
α = 87.082 (8)°	*T* = 173 K
β = 88.326 (8)°	Plate, colourless
γ = 83.988 (8)°	0.40 × 0.15 × 0.15 mm
*V* = 1784.3 (3) Å^3^	

### Data collection

**Table:**

Stoe IPDS II CCD diffractometer	5925 reflections with *I* > 2σ(*I*)
Radiation source: fine-focus sealed tube	*R*_int_ = 0.093
graphite	θ_max_ = 26.4°, θ_min_ = 3.2°
φ scans	*h* = −11→9
25481 measured reflections	*k* = −17→17
7264 independent reflections	*l* = −17→17

### Refinement

**Table:**

Refinement on *F*^2^	Secondary atom site location: difference Fourier map
Least-squares matrix: full	Hydrogen site location: inferred from neighbouring sites
*R*[*F*^2^ > 2σ(*F*^2^)] = 0.048	H atoms treated by a mixture of independent and constrained refinement
*wR*(*F*^2^) = 0.105	*w* = 1/[σ^2^(*F*_o_^2^) + (0.0217*P*)^2^ + 0.9275*P*] where *P* = (*F*_o_^2^ + 2*F*_c_^2^)/3
*S* = 1.13	(Δ/σ)_max_ = 0.001
7264 reflections	Δρ_max_ = 0.43 e Å^−^^3^
464 parameters	Δρ_min_ = −0.27 e Å^−^^3^
0 restraints	Extinction correction: *SHELXL97* (Sheldrick, 2008), Fc^*^=kFc[1+0.001xFc^2^λ^3^/sin(2θ)]^-1/4^
Primary atom site location: structure-invariant direct methods	Extinction coefficient: 0.0089 (11)

### Special details

**Table:**

Geometry. All e.s.d.'s (except the e.s.d. in the dihedral angle between two l.s. planes) are estimated using the full covariance matrix. The cell e.s.d.'s are taken into account individually in the estimation of e.s.d.'s in distances, angles and torsion angles; correlations between e.s.d.'s in cell parameters are only used when they are defined by crystal symmetry. An approximate (isotropic) treatment of cell e.s.d.'s is used for estimating e.s.d.'s involving l.s. planes.
Refinement. Refinement of *F*^2^ against ALL reflections. The weighted *R*-factor *wR* and goodness of fit *S* are based on *F*^2^, conventional *R*-factors *R* are based on *F*, with *F* set to zero for negative *F*^2^. The threshold expression of *F*^2^ > σ(*F*^2^) is used only for calculating *R*-factors(gt) *etc*. and is not relevant to the choice of reflections for refinement. *R*-factors based on *F*^2^ are statistically about twice as large as those based on *F*, and *R*- factors based on ALL data will be even larger.

### Fractional atomic coordinates and isotropic or equivalent isotropic displacement parameters (Å^2^)

**Table:**

	*x*	*y*	*z*	*U*_iso_*/*U*_eq_	
S1	0.43909 (5)	0.75074 (4)	0.39894 (3)	0.02465 (13)	
N2	0.62257 (18)	0.61645 (11)	0.67491 (11)	0.0239 (3)	
N5	0.44232 (18)	0.57763 (11)	0.76972 (11)	0.0239 (3)	
O11	0.39575 (19)	0.65641 (11)	0.38435 (10)	0.0380 (4)	
O12	0.37379 (19)	0.82319 (12)	0.33265 (10)	0.0392 (4)	
O13	0.60130 (17)	0.75129 (12)	0.40749 (11)	0.0407 (4)	
C1	0.5287 (2)	0.55055 (14)	0.69578 (13)	0.0236 (4)	
H1	0.5239	0.4939	0.6636	0.028*	
C3	0.5962 (2)	0.68882 (14)	0.73746 (14)	0.0274 (4)	
C4	0.4838 (2)	0.66389 (14)	0.79749 (14)	0.0271 (4)	
C51	0.3196 (2)	0.52557 (15)	0.81440 (14)	0.0278 (4)	
H51	0.2354	0.5736	0.8340	0.033*	
C11	0.3598 (2)	0.78283 (14)	0.51005 (13)	0.0243 (4)	
C12	0.2803 (2)	0.72031 (15)	0.56465 (13)	0.0275 (4)	
H12	0.2693	0.6588	0.5437	0.033*	
C13	0.2164 (2)	0.74776 (16)	0.65032 (14)	0.0332 (5)	
H13	0.1614	0.7051	0.6876	0.040*	
C14	0.2328 (3)	0.83630 (17)	0.68102 (15)	0.0406 (6)	
H14	0.1885	0.8549	0.7393	0.049*	
C15	0.3137 (3)	0.89853 (17)	0.62733 (17)	0.0472 (6)	
H15	0.3263	0.9593	0.6494	0.057*	
C16	0.3769 (3)	0.87242 (16)	0.54092 (16)	0.0376 (5)	
H16	0.4312	0.9155	0.5036	0.045*	
C21	0.7465 (2)	0.61059 (15)	0.60282 (14)	0.0287 (4)	
H21	0.7546	0.6761	0.5748	0.034*	
C22	0.8952 (3)	0.57618 (19)	0.64945 (17)	0.0431 (6)	
H22A	0.9131	0.6189	0.6988	0.065*	
H22B	0.9783	0.5758	0.6028	0.065*	
H22C	0.8900	0.5116	0.6768	0.065*	
C23	0.7120 (3)	0.54755 (16)	0.52511 (15)	0.0357 (5)	
H23A	0.6156	0.5723	0.4969	0.054*	
H23B	0.7049	0.4827	0.5510	0.054*	
H23C	0.7934	0.5470	0.4773	0.054*	
C31	0.6752 (3)	0.77647 (17)	0.72980 (19)	0.0374 (5)	
H60	0.655 (3)	0.811 (2)	0.673 (2)	0.051 (8)*	
H61	0.783 (3)	0.7619 (19)	0.7328 (18)	0.049 (7)*	
H62	0.652 (3)	0.815 (2)	0.784 (2)	0.061 (8)*	
C41	0.4125 (3)	0.71378 (18)	0.87908 (17)	0.0367 (5)	
H63	0.437 (3)	0.681 (2)	0.936 (2)	0.054 (8)*	
H64	0.309 (4)	0.723 (2)	0.877 (2)	0.058 (8)*	
H65	0.451 (3)	0.777 (2)	0.8844 (19)	0.058 (8)*	
C53	0.2571 (3)	0.46273 (18)	0.74510 (16)	0.0386 (5)	
H53A	0.2201	0.5018	0.6904	0.058*	
H53B	0.1735	0.4313	0.7748	0.058*	
H53C	0.3376	0.4145	0.7254	0.058*	
C52	0.3776 (3)	0.46925 (17)	0.90138 (15)	0.0392 (5)	
H52A	0.4158	0.5124	0.9444	0.059*	
H52B	0.4596	0.4211	0.8838	0.059*	
H52C	0.2947	0.4379	0.9322	0.059*	
S2	−0.08093 (6)	0.73424 (4)	−0.07445 (3)	0.02813 (13)	
O21	−0.19080 (19)	0.66586 (13)	−0.07226 (12)	0.0459 (4)	
O22	0.02063 (18)	0.72912 (14)	−0.15586 (10)	0.0472 (4)	
O23	−0.14735 (18)	0.83069 (11)	−0.05920 (11)	0.0384 (4)	
N02	0.75192 (18)	0.90033 (11)	0.17822 (11)	0.0247 (3)	
N05	0.90956 (18)	0.87415 (12)	0.29144 (12)	0.0276 (4)	
C01	0.7870 (2)	0.84590 (14)	0.25474 (14)	0.0268 (4)	
H01	0.7331	0.7951	0.2791	0.032*	
C03	0.8562 (2)	0.96711 (13)	0.16604 (14)	0.0252 (4)	
C04	0.9551 (2)	0.95119 (14)	0.23694 (14)	0.0271 (4)	
C011	0.0379 (2)	0.70085 (14)	0.02239 (13)	0.0253 (4)	
C012	0.0905 (3)	0.60555 (15)	0.03882 (15)	0.0350 (5)	
H012	0.0623	0.5593	−0.0013	0.042*	
C013	0.1834 (3)	0.57763 (17)	0.11302 (17)	0.0419 (6)	
H013	0.2173	0.5123	0.1246	0.050*	
C014	0.2274 (3)	0.64522 (18)	0.17066 (15)	0.0394 (5)	
H014	0.2915	0.6262	0.2217	0.047*	
C015	0.1777 (3)	0.74003 (16)	0.15365 (14)	0.0343 (5)	
H015	0.2090	0.7863	0.1927	0.041*	
C016	0.0826 (2)	0.76818 (15)	0.08013 (13)	0.0281 (4)	
H016	0.0480	0.8335	0.0692	0.034*	
C031	0.8501 (3)	1.03796 (16)	0.08522 (16)	0.0321 (5)	
H54	0.856 (3)	1.0048 (17)	0.0264 (17)	0.034 (6)*	
H55	0.934 (3)	1.0736 (19)	0.0873 (17)	0.041 (7)*	
H56	0.758 (3)	1.083 (2)	0.0841 (19)	0.053 (8)*	
C021	0.6220 (2)	0.89358 (16)	0.11676 (14)	0.0309 (5)	
H021	0.6537	0.9119	0.0513	0.037*	
C022	0.4900 (3)	0.96410 (19)	0.14589 (18)	0.0433 (6)	
H02A	0.5224	1.0283	0.1435	0.065*	
H02B	0.4563	0.9472	0.2098	0.065*	
H02C	0.4061	0.9623	0.1032	0.065*	
C023	0.5808 (3)	0.79234 (17)	0.11832 (16)	0.0399 (5)	
H02D	0.6694	0.7499	0.0990	0.060*	
H02E	0.4982	0.7885	0.0752	0.060*	
H02F	0.5481	0.7731	0.1818	0.060*	
C041	1.0886 (3)	1.00083 (18)	0.25941 (19)	0.0377 (5)	
H57	1.180 (3)	0.9550 (19)	0.2658 (18)	0.046 (7)*	
H58	1.071 (3)	1.033 (2)	0.323 (2)	0.053 (8)*	
H59	1.106 (3)	1.049 (2)	0.212 (2)	0.052 (8)*	
C051	0.9840 (2)	0.83105 (16)	0.37810 (16)	0.0349 (5)	
H051	1.0931	0.8432	0.3743	0.042*	
C052	0.9770 (3)	0.72410 (16)	0.38417 (16)	0.0376 (5)	
H05A	1.0251	0.6963	0.3280	0.056*	
H05B	0.8709	0.7105	0.3888	0.056*	
H05C	1.0306	0.6964	0.4396	0.056*	
C053	0.9122 (3)	0.87818 (19)	0.46308 (17)	0.0491 (6)	
H05D	0.9196	0.9468	0.4565	0.074*	
H05E	0.9652	0.8518	0.5192	0.074*	
H05F	0.8053	0.8665	0.4687	0.074*	

### Atomic displacement parameters (Å^2^)

**Table:**

	*U*^11^	*U*^22^	*U*^33^	*U*^12^	*U*^13^	*U*^23^
S1	0.0242 (2)	0.0278 (3)	0.0214 (2)	−0.00192 (19)	0.00511 (18)	−0.00097 (18)
N2	0.0237 (8)	0.0233 (8)	0.0245 (8)	−0.0002 (7)	−0.0032 (6)	−0.0021 (6)
N5	0.0248 (8)	0.0244 (8)	0.0221 (8)	0.0006 (7)	−0.0012 (6)	−0.0038 (6)
O11	0.0511 (10)	0.0331 (8)	0.0314 (8)	−0.0104 (7)	0.0122 (7)	−0.0108 (6)
O12	0.0466 (9)	0.0425 (9)	0.0258 (7)	0.0046 (7)	0.0014 (7)	0.0046 (6)
O13	0.0248 (8)	0.0553 (10)	0.0410 (9)	−0.0039 (7)	0.0079 (7)	0.0037 (8)
C1	0.0247 (9)	0.0236 (9)	0.0221 (9)	0.0001 (8)	−0.0025 (7)	−0.0030 (7)
C3	0.0287 (10)	0.0232 (10)	0.0304 (10)	0.0012 (8)	−0.0078 (8)	−0.0055 (8)
C4	0.0271 (10)	0.0252 (10)	0.0290 (10)	0.0011 (8)	−0.0056 (8)	−0.0061 (8)
C51	0.0242 (10)	0.0321 (11)	0.0270 (10)	−0.0012 (8)	0.0027 (8)	−0.0049 (8)
C11	0.0241 (9)	0.0272 (10)	0.0209 (9)	0.0017 (8)	−0.0004 (7)	−0.0027 (7)
C12	0.0268 (10)	0.0306 (11)	0.0245 (9)	−0.0004 (8)	0.0006 (8)	−0.0007 (8)
C13	0.0323 (11)	0.0422 (13)	0.0228 (10)	0.0053 (10)	0.0033 (8)	0.0019 (9)
C14	0.0489 (14)	0.0454 (14)	0.0235 (10)	0.0134 (11)	0.0029 (9)	−0.0046 (9)
C15	0.0678 (17)	0.0313 (12)	0.0421 (13)	0.0043 (12)	−0.0037 (12)	−0.0166 (10)
C16	0.0499 (14)	0.0291 (11)	0.0342 (11)	−0.0050 (10)	0.0000 (10)	−0.0044 (9)
C21	0.0320 (11)	0.0258 (10)	0.0281 (10)	−0.0045 (8)	0.0055 (8)	0.0002 (8)
C22	0.0278 (11)	0.0550 (15)	0.0453 (13)	−0.0005 (11)	0.0034 (10)	−0.0026 (11)
C23	0.0460 (13)	0.0342 (12)	0.0271 (10)	−0.0067 (10)	0.0076 (9)	−0.0030 (9)
C31	0.0403 (14)	0.0303 (12)	0.0431 (14)	−0.0077 (10)	−0.0067 (11)	−0.0055 (10)
C41	0.0414 (14)	0.0351 (13)	0.0336 (12)	0.0010 (11)	0.0010 (10)	−0.0125 (10)
C53	0.0351 (12)	0.0487 (14)	0.0345 (11)	−0.0147 (10)	0.0016 (9)	−0.0057 (10)
C52	0.0455 (13)	0.0417 (13)	0.0303 (11)	−0.0058 (11)	−0.0010 (10)	0.0038 (10)
S2	0.0270 (3)	0.0364 (3)	0.0221 (2)	−0.0092 (2)	0.00347 (19)	−0.0028 (2)
O21	0.0448 (10)	0.0489 (10)	0.0483 (10)	−0.0236 (8)	−0.0089 (8)	−0.0003 (8)
O22	0.0375 (9)	0.0792 (13)	0.0236 (7)	−0.0018 (9)	0.0073 (7)	−0.0025 (8)
O23	0.0381 (9)	0.0389 (9)	0.0374 (8)	−0.0014 (7)	−0.0027 (7)	0.0012 (7)
N02	0.0255 (8)	0.0246 (8)	0.0239 (8)	−0.0040 (7)	0.0056 (6)	−0.0010 (6)
N05	0.0230 (8)	0.0285 (9)	0.0311 (9)	−0.0047 (7)	0.0015 (7)	0.0027 (7)
C01	0.0248 (10)	0.0272 (10)	0.0284 (10)	−0.0064 (8)	0.0028 (8)	0.0024 (8)
C03	0.0257 (10)	0.0200 (9)	0.0297 (10)	−0.0038 (8)	0.0094 (8)	−0.0036 (8)
C04	0.0261 (10)	0.0208 (9)	0.0342 (10)	−0.0032 (8)	0.0084 (8)	−0.0023 (8)
C011	0.0265 (10)	0.0295 (10)	0.0207 (9)	−0.0087 (8)	0.0063 (7)	−0.0014 (8)
C012	0.0434 (13)	0.0270 (11)	0.0363 (11)	−0.0103 (9)	0.0039 (10)	−0.0057 (9)
C013	0.0486 (14)	0.0306 (12)	0.0443 (13)	0.0013 (10)	0.0031 (11)	0.0067 (10)
C014	0.0369 (12)	0.0496 (14)	0.0296 (11)	0.0035 (11)	0.0006 (9)	0.0017 (10)
C015	0.0366 (12)	0.0400 (12)	0.0271 (10)	−0.0043 (10)	0.0018 (9)	−0.0087 (9)
C016	0.0332 (11)	0.0263 (10)	0.0248 (9)	−0.0026 (8)	0.0060 (8)	−0.0064 (8)
C031	0.0363 (12)	0.0275 (11)	0.0326 (11)	−0.0067 (10)	0.0091 (9)	0.0003 (9)
C021	0.0307 (11)	0.0398 (12)	0.0233 (9)	−0.0106 (9)	0.0011 (8)	0.0005 (8)
C022	0.0301 (12)	0.0508 (15)	0.0470 (14)	0.0000 (11)	−0.0022 (10)	0.0085 (11)
C023	0.0455 (13)	0.0461 (14)	0.0320 (11)	−0.0218 (11)	0.0046 (10)	−0.0076 (10)
C041	0.0290 (11)	0.0315 (12)	0.0532 (15)	−0.0081 (10)	0.0010 (10)	0.0003 (11)
C051	0.0255 (10)	0.0389 (12)	0.0404 (12)	−0.0082 (9)	−0.0064 (9)	0.0098 (10)
C052	0.0310 (11)	0.0379 (12)	0.0416 (12)	−0.0001 (9)	0.0003 (9)	0.0110 (10)
C053	0.0626 (17)	0.0488 (15)	0.0364 (13)	−0.0044 (13)	−0.0163 (12)	−0.0019 (11)

### Geometric parameters (Å, °)

**Table:**

S1—O12	1.4478 (15)	S2—O21	1.4420 (16)
S1—O13	1.4483 (16)	S2—O22	1.4523 (15)
S1—O11	1.4537 (16)	S2—O23	1.4532 (17)
S1—C11	1.7816 (19)	S2—C011	1.781 (2)
N2—C1	1.331 (3)	N02—C01	1.332 (2)
N2—C3	1.392 (2)	N02—C03	1.391 (2)
N2—C21	1.484 (2)	N02—C021	1.484 (3)
N5—C1	1.337 (2)	N05—C01	1.328 (3)
N5—C4	1.391 (3)	N05—C04	1.395 (2)
N5—C51	1.489 (3)	N05—C051	1.495 (3)
C1—H1	0.9500	C01—H01	0.9500
C3—C4	1.360 (3)	C03—C04	1.356 (3)
C3—C31	1.485 (3)	C03—C031	1.490 (3)
C4—C41	1.487 (3)	C04—C041	1.490 (3)
C51—C52	1.517 (3)	C011—C016	1.388 (3)
C51—C53	1.519 (3)	C011—C012	1.391 (3)
C51—H51	1.0000	C012—C013	1.380 (3)
C11—C12	1.385 (3)	C012—H012	0.9500
C11—C16	1.388 (3)	C013—C014	1.387 (4)
C12—C13	1.392 (3)	C013—H013	0.9500
C12—H12	0.9500	C014—C015	1.379 (3)
C13—C14	1.372 (3)	C014—H014	0.9500
C13—H13	0.9500	C015—C016	1.385 (3)
C14—C15	1.383 (4)	C015—H015	0.9500
C14—H14	0.9500	C016—H016	0.9500
C15—C16	1.394 (3)	C031—H54	0.98 (2)
C15—H15	0.9500	C031—H55	0.94 (3)
C16—H16	0.9500	C031—H56	0.98 (3)
C21—C23	1.518 (3)	C021—C023	1.514 (3)
C21—C22	1.519 (3)	C021—C022	1.520 (3)
C21—H21	1.0000	C021—H021	1.0000
C22—H22A	0.9800	C022—H02A	0.9800
C22—H22B	0.9800	C022—H02B	0.9800
C22—H22C	0.9800	C022—H02C	0.9800
C23—H23A	0.9800	C023—H02D	0.9800
C23—H23B	0.9800	C023—H02E	0.9800
C23—H23C	0.9800	C023—H02F	0.9800
C31—H60	0.94 (3)	C041—H57	0.98 (3)
C31—H61	0.96 (3)	C041—H58	1.04 (3)
C31—H62	0.97 (3)	C041—H59	0.96 (3)
C41—H63	0.94 (3)	C051—C053	1.510 (3)
C41—H64	0.92 (3)	C051—C052	1.519 (3)
C41—H65	1.00 (3)	C051—H051	1.0000
C53—H53A	0.9800	C052—H05A	0.9800
C53—H53B	0.9800	C052—H05B	0.9800
C53—H53C	0.9800	C052—H05C	0.9800
C52—H52A	0.9800	C053—H05D	0.9800
C52—H52B	0.9800	C053—H05E	0.9800
C52—H52C	0.9800	C053—H05F	0.9800
			
O12—S1—O13	113.12 (10)	O21—S2—O22	113.55 (11)
O12—S1—O11	113.52 (10)	O21—S2—O23	113.47 (10)
O13—S1—O11	112.59 (10)	O22—S2—O23	112.47 (10)
O12—S1—C11	105.44 (9)	O21—S2—C011	105.64 (10)
O13—S1—C11	105.24 (9)	O22—S2—C011	104.85 (9)
O11—S1—C11	106.02 (9)	O23—S2—C011	105.90 (9)
C1—N2—C3	109.23 (16)	C01—N02—C03	108.49 (17)
C1—N2—C21	126.08 (16)	C01—N02—C021	126.48 (17)
C3—N2—C21	124.44 (17)	C03—N02—C021	125.02 (16)
C1—N5—C4	108.81 (16)	C01—N05—C04	108.89 (17)
C1—N5—C51	125.99 (17)	C01—N05—C051	125.15 (17)
C4—N5—C51	125.19 (16)	C04—N05—C051	125.95 (17)
N2—C1—N5	108.40 (17)	N05—C01—N02	108.90 (17)
N2—C1—H1	125.8	N05—C01—H01	125.6
N5—C1—H1	125.8	N02—C01—H01	125.6
C4—C3—N2	106.58 (17)	C04—C03—N02	107.27 (17)
C4—C3—C31	130.40 (19)	C04—C03—C031	130.82 (19)
N2—C3—C31	122.91 (19)	N02—C03—C031	121.89 (19)
C3—C4—N5	106.98 (17)	C03—C04—N05	106.45 (17)
C3—C4—C41	129.8 (2)	C03—C04—C041	130.81 (19)
N5—C4—C41	123.20 (19)	N05—C04—C041	122.7 (2)
N5—C51—C52	110.01 (17)	C016—C011—C012	119.32 (19)
N5—C51—C53	110.63 (16)	C016—C011—S2	121.44 (16)
C52—C51—C53	112.06 (19)	C012—C011—S2	119.21 (16)
N5—C51—H51	108.0	C013—C012—C011	120.5 (2)
C52—C51—H51	108.0	C013—C012—H012	119.7
C53—C51—H51	108.0	C011—C012—H012	119.7
C12—C11—C16	120.16 (19)	C012—C013—C014	119.9 (2)
C12—C11—S1	120.90 (15)	C012—C013—H013	120.1
C16—C11—S1	118.93 (16)	C014—C013—H013	120.1
C11—C12—C13	119.9 (2)	C015—C014—C013	119.8 (2)
C11—C12—H12	120.1	C015—C014—H014	120.1
C13—C12—H12	120.1	C013—C014—H014	120.1
C14—C13—C12	120.2 (2)	C014—C015—C016	120.5 (2)
C14—C13—H13	119.9	C014—C015—H015	119.8
C12—C13—H13	119.9	C016—C015—H015	119.8
C13—C14—C15	120.2 (2)	C015—C016—C011	120.0 (2)
C13—C14—H14	119.9	C015—C016—H016	120.0
C15—C14—H14	119.9	C011—C016—H016	120.0
C14—C15—C16	120.3 (2)	C03—C031—H54	109.7 (13)
C14—C15—H15	119.9	C03—C031—H55	108.9 (15)
C16—C15—H15	119.9	H54—C031—H55	109 (2)
C11—C16—C15	119.3 (2)	C03—C031—H56	114.2 (15)
C11—C16—H16	120.3	H54—C031—H56	108 (2)
C15—C16—H16	120.3	H55—C031—H56	108 (2)
N2—C21—C23	111.13 (17)	N02—C021—C023	110.53 (18)
N2—C21—C22	109.09 (17)	N02—C021—C022	109.35 (18)
C23—C21—C22	112.18 (19)	C023—C021—C022	112.72 (19)
N2—C21—H21	108.1	N02—C021—H021	108.0
C23—C21—H21	108.1	C023—C021—H021	108.0
C22—C21—H21	108.1	C022—C021—H021	108.0
C21—C22—H22A	109.5	C021—C022—H02A	109.5
C21—C22—H22B	109.5	C021—C022—H02B	109.5
H22A—C22—H22B	109.5	H02A—C022—H02B	109.5
C21—C22—H22C	109.5	C021—C022—H02C	109.5
H22A—C22—H22C	109.5	H02A—C022—H02C	109.5
H22B—C22—H22C	109.5	H02B—C022—H02C	109.5
C21—C23—H23A	109.5	C021—C023—H02D	109.5
C21—C23—H23B	109.5	C021—C023—H02E	109.5
H23A—C23—H23B	109.5	H02D—C023—H02E	109.5
C21—C23—H23C	109.5	C021—C023—H02F	109.5
H23A—C23—H23C	109.5	H02D—C023—H02F	109.5
H23B—C23—H23C	109.5	H02E—C023—H02F	109.5
C3—C31—H60	111.4 (17)	C04—C041—H57	110.4 (15)
C3—C31—H61	111.4 (16)	C04—C041—H58	110.7 (15)
H60—C31—H61	107 (2)	H57—C041—H58	107 (2)
C3—C31—H62	111.2 (17)	C04—C041—H59	110.7 (17)
H60—C31—H62	113 (2)	H57—C041—H59	110 (2)
H61—C31—H62	102 (2)	H58—C041—H59	108 (2)
C4—C41—H63	112.5 (18)	N05—C051—C053	110.09 (18)
C4—C41—H64	113.4 (19)	N05—C051—C052	110.38 (18)
H63—C41—H64	107 (2)	C053—C051—C052	112.08 (19)
C4—C41—H65	111.2 (17)	N05—C051—H051	108.1
H63—C41—H65	104 (2)	C053—C051—H051	108.1
H64—C41—H65	108 (2)	C052—C051—H051	108.1
C51—C53—H53A	109.5	C051—C052—H05A	109.5
C51—C53—H53B	109.5	C051—C052—H05B	109.5
H53A—C53—H53B	109.5	H05A—C052—H05B	109.5
C51—C53—H53C	109.5	C051—C052—H05C	109.5
H53A—C53—H53C	109.5	H05A—C052—H05C	109.5
H53B—C53—H53C	109.5	H05B—C052—H05C	109.5
C51—C52—H52A	109.5	C051—C053—H05D	109.5
C51—C52—H52B	109.5	C051—C053—H05E	109.5
H52A—C52—H52B	109.5	H05D—C053—H05E	109.5
C51—C52—H52C	109.5	C051—C053—H05F	109.5
H52A—C52—H52C	109.5	H05D—C053—H05F	109.5
H52B—C52—H52C	109.5	H05E—C053—H05F	109.5
			
C3—N2—C1—N5	0.0 (2)	C04—N05—C01—N02	0.8 (2)
C21—N2—C1—N5	174.43 (16)	C051—N05—C01—N02	179.71 (18)
C4—N5—C1—N2	−0.5 (2)	C03—N02—C01—N05	−0.6 (2)
C51—N5—C1—N2	178.39 (16)	C021—N02—C01—N05	−179.25 (17)
C1—N2—C3—C4	0.5 (2)	C01—N02—C03—C04	0.2 (2)
C21—N2—C3—C4	−174.03 (17)	C021—N02—C03—C04	178.88 (17)
C1—N2—C3—C31	−175.93 (19)	C01—N02—C03—C031	178.83 (18)
C21—N2—C3—C31	9.5 (3)	C021—N02—C03—C031	−2.5 (3)
N2—C3—C4—N5	−0.8 (2)	N02—C03—C04—N05	0.2 (2)
C31—C3—C4—N5	175.3 (2)	C031—C03—C04—N05	−178.2 (2)
N2—C3—C4—C41	178.8 (2)	N02—C03—C04—C041	−179.7 (2)
C31—C3—C4—C41	−5.2 (4)	C031—C03—C04—C041	1.9 (4)
C1—N5—C4—C3	0.8 (2)	C01—N05—C04—C03	−0.6 (2)
C51—N5—C4—C3	−178.09 (17)	C051—N05—C04—C03	−179.54 (18)
C1—N5—C4—C41	−178.78 (19)	C01—N05—C04—C041	179.32 (19)
C51—N5—C4—C41	2.3 (3)	C051—N05—C04—C041	0.4 (3)
C1—N5—C51—C52	99.9 (2)	O21—S2—C011—C016	−136.91 (17)
C4—N5—C51—C52	−81.4 (2)	O22—S2—C011—C016	102.86 (18)
C1—N5—C51—C53	−24.4 (3)	O23—S2—C011—C016	−16.26 (18)
C4—N5—C51—C53	154.31 (19)	O21—S2—C011—C012	45.03 (19)
O12—S1—C11—C12	122.81 (17)	O22—S2—C011—C012	−75.20 (18)
O13—S1—C11—C12	−117.36 (17)	O23—S2—C011—C012	165.67 (16)
O11—S1—C11—C12	2.15 (19)	C016—C011—C012—C013	1.5 (3)
O12—S1—C11—C16	−56.32 (19)	S2—C011—C012—C013	179.62 (17)
O13—S1—C11—C16	63.52 (19)	C011—C012—C013—C014	−1.3 (3)
O11—S1—C11—C16	−176.98 (17)	C012—C013—C014—C015	0.1 (3)
C16—C11—C12—C13	0.5 (3)	C013—C014—C015—C016	0.8 (3)
S1—C11—C12—C13	−178.59 (15)	C014—C015—C016—C011	−0.6 (3)
C11—C12—C13—C14	−0.4 (3)	C012—C011—C016—C015	−0.5 (3)
C12—C13—C14—C15	−0.4 (3)	S2—C011—C016—C015	−178.60 (15)
C13—C14—C15—C16	1.1 (4)	C01—N02—C021—C023	−29.3 (3)
C12—C11—C16—C15	0.1 (3)	C03—N02—C021—C023	152.33 (18)
S1—C11—C16—C15	179.28 (18)	C01—N02—C021—C022	95.4 (2)
C14—C15—C16—C11	−1.0 (4)	C03—N02—C021—C022	−83.0 (2)
C1—N2—C21—C23	27.3 (3)	C01—N05—C051—C053	−87.7 (3)
C3—N2—C21—C23	−159.08 (18)	C04—N05—C051—C053	91.0 (2)
C1—N2—C21—C22	−96.9 (2)	C01—N05—C051—C052	36.5 (3)
C3—N2—C21—C22	76.7 (2)	C04—N05—C051—C052	−144.70 (19)

### Hydrogen-bond geometry (Å, °)

**Table:**

*D*—H···*A*	*D*—H	H···*A*	*D*···*A*	*D*—H···*A*
C1—H1···O11^i^	0.95	2.30	3.198 (3)	157
C01—H01···O13	0.95	2.25	3.040 (3)	141
C021—H021···O23^ii^	1.00	2.56	3.297 (3)	131
C022—H02B···O12	0.98	2.57	3.452 (3)	150
C13—H13···O22^iii^	0.95	2.55	3.245 (2)	130
C052—H05B···O13	0.98	2.41	3.323 (3)	155
C23—H23B···O11^i^	0.98	2.38	3.324 (3)	162

Symmetry codes: (i) −*x*+1, −*y*+1, −*z*+1; (ii) *x*+1, *y*, *z*; (iii) *x*, *y*, *z*+1.
